# Wearable Patch ECG monitors and transesophageal electrophysiological study for diagnosing palpitations of unknown origin

**DOI:** 10.3389/fcvm.2024.1469108

**Published:** 2024-11-13

**Authors:** Ruike Yang, Lihong Yang, Qiang Zhang, Shuhui Wang, Jinyi Xu

**Affiliations:** ^1^ Department of Cardiopulmonary Function, Henan Provincial People’s Hospital, People’s Hospital of Zhengzhou University, Zhengzhou, Henan, China; ^2^ Department of Cardiology, The Second Affiliated Hospital of Zhengzhou University, Zhengzhou, Henan, China; ^3^ International Medical Center, Henan Provincial People’s Hospital, People’s Hospital of Zhengzhou University, Zhengzhou, Henan, China

**Keywords:** palpitations of unknown origin, transesophageal electrophysiological study, wearable adhesive Patch ECG monitors, diagnosis, cardiac electrophysiology

## Abstract

**Objective:**

To analyze the application value of wearable adhesive Patch ECG monitors combined with transesophageal electrophysiological study (TEPS) in the diagnosis of palpitations of unknown origin.

**Methods:**

This was a retrospective study of patients with suspected arrhythmia who were admitted to Henan Provincial People's Hospital between October 2021 and July 2023 due to recurrent paroxysmal palpitations of unknown origin, with or without accompanying symptoms such as dizziness, amaurosis, and syncope. All patients underwent TEPS. Those who did not exhibit arrhythmia during the TEPS were selected for Patch ECG monitoring, which lasted several weeks (depending on the duration of symptom capture). The results of TEPS, Patch ECG monitors, and clinical diagnoses were observed and recorded. Sensitivity, specificity, accuracy, positive predictive value, and negative predictive value (NPV) for diagnosing palpitations of unknown origins was analyzed based on clinical diagnostic outcomes for (1) TEPS alone, (2) Patch ECG monitoring in patients with negative TEPS results, and (3) the combination of both methods.

**Results:**

A total of 569 patients were included in this study. The TEPS results exhibited that 227 of the 569 patients did not detect arrhythmias and 342 detected arrhythmias. Of the 569 patients, 102 refused to undergo Patch ECG monitors, and 467 patients completed the entire study process. Among them, 379 cases (66.61%) were clinically diagnosed as arrhythmias. TEPS shows good performance in most evaluation indices except NPV (69.60%, 95% CI, 61.54%–77.66%). The combined diagnosis was strongly consistent with clinical diagnosis. The accuracy, sensitivity, and NPV of TEPS combined with Patch ECG monitors in the diagnosis of palpitations of unknown origin were significantly higher than those of TEPS alone.

**Conclusion:**

Wearable adhesive patch ECG monitors combined with TEPS can enhance the diagnostic efficiency of palpitations of unknown origin.

## 1 Introduction

Palpitations are among the most common subjective symptoms of patients presenting to primary care providers and cardiologists, characterized by an individual's subjective experience of an unpleasant awareness of the heart beating forceful, rapidly, regularly, or irregularly (1). Patients may also describe this sensation as a rapid fluttering or pounding in the chest or neck areas. The experience of palpitations can vary significantly among patients, ranging from a mild, transient sensation to a more intense and sustained feeling of discomfort. Some may even perceive their heart as pounding against their chest wall, creating a sensation that can be both alarming and disruptive to their daily activities.

The most common causes of palpitations include heart disease, physical diseases with endocrine and metabolic abnormalities, mental disorders, and drug side effects (2). For certain patients experiencing palpitations, clinicians can ascertain the underlying cause through a comprehensive medical history assessment, meticulous physical examination, 12-lead ECG, Holter monitors and pertinent laboratory investigations. However, in patients with an undetermined etiology, establishing a diagnosis poses considerable challenges (1, 3). When utilizing Rest 12-lead ECG and Holter monitors, clinicians can encounter difficulties in precisely diagnosing the underlying cause of palpitations with an unknown source, primarily due to constraints imposed by their limited observation windows. Wearable adhesive Patch ECG monitors, based on 24-h Holter monitors, are designed for long-term patient monitoring using a compact, waterproof, and high-capacity ECG recorder. This allows the detection of occasional abnormal electrical activity, providing an accurate and reliable foundation for diagnosing various arrhythmias (4–6).

Transesophageal electrophysiological study (TEPS) is a well-established procedure that employs esophageal pacing leads to indirectly stimulate and monitor the heart's electrical activity. Pacing leads are inserted through either the nose or mouth and positioned in the esophagus, eliminating the need for skin punctures, blood vessel invasion, or direct heart access. This method offers a relatively non-invasive alternative for pacing and assessing the heart without the requirement of fluoroscopy, strict sterile precautions, or cardiac catheterization (7–10). TEPS can not only induce tachycardia by different stimulation methods and evaluate the risk stratification of pre-excitation bypass, but also effectively terminate the onset of supraventricular tachycardia (SVT), atrial flutter, and avoid aggravation and deterioration of the original arrhythmia (11–20). The 2019 ESC Guidelines for managing patients with supraventricular tachycardia endorses the use of esophageal electrocardiogram for the differential diagnosis of narrow QRS tachycardia (19). As a lower-cost diagnostic and treatment option with fewer operational requirements, it is often utilized in many primary healthcare institutions and developing countries (9–11, 13–16, 20).

Currently, Patch ECG monitors and TEPS have been applied separately in the diagnosis of palpitations of unknown origin, and they have been proven to have certain diagnostic efficacy (21, 22). However, there are still missed diagnoses and misdiagnoses when applied alone. Combining these methods could improve the diagnostic efficiency of palpitations of unknown origin, but there are few relevant studies. Therefore, this study analyzed the application value of Patch ECG monitors combined with TEPS for the diagnosis of palpitations of unknown origin, aiming to provide a reference for the improvement of diagnostic methods.

## 2 Data and methods

### 2.1 General data

Patients with unexplained palpitations who were recommended for TEPS and Patch ECG monitors based on clinical evaluation between October 2021 and July 2023 were included in this study. The criteria for inclusion and exclusion were carefully established to ensure accuracy.

The inclusion criteria were (1) presence of recurrent paroxysmal palpitations, with or without accompanying symptoms such as dizziness, amaurosis, syncope, or suspicion of an underlying arrhythmia; (2) abrupt manifestation and sudden or gradual cessation of symptoms lasting from seconds to minutes or even hours (onset and relief may occur with or without a consistent triggering factor); (3) completion of initial comprehensive evaluation yielding negative results, including a thorough review of medical history, detailed physical examination, ECG, cardiac ultrasound imaging, and various laboratory tests ruling out other potential causes; (4) absence of antiarrhythmic drugs or other medications that could affect the diagnosis; and (5) obtaining written informed consent.

Exclusion criteria were (1) presence of significant esophageal conditions or severe nasal pathologies; (2) severe myocardial ischemia, cardiac enlargement, or heart failure; (3) severe hypertension, defined as blood pressure exceeding 200/110 mmHg; (4) refusal to continue using the Patch ECG monitors after completing the TEPS process; (5) inability to obtain valid data from Patch ECG monitors due to various factors such as skin allergies, intense physical exertion, detachment of patches, or occurrence of severe non-cardiac adverse events requiring immediate medical attention during the monitoring period; and (6) lack of follow-up records for accurate diagnosis.

**Figure 1:**
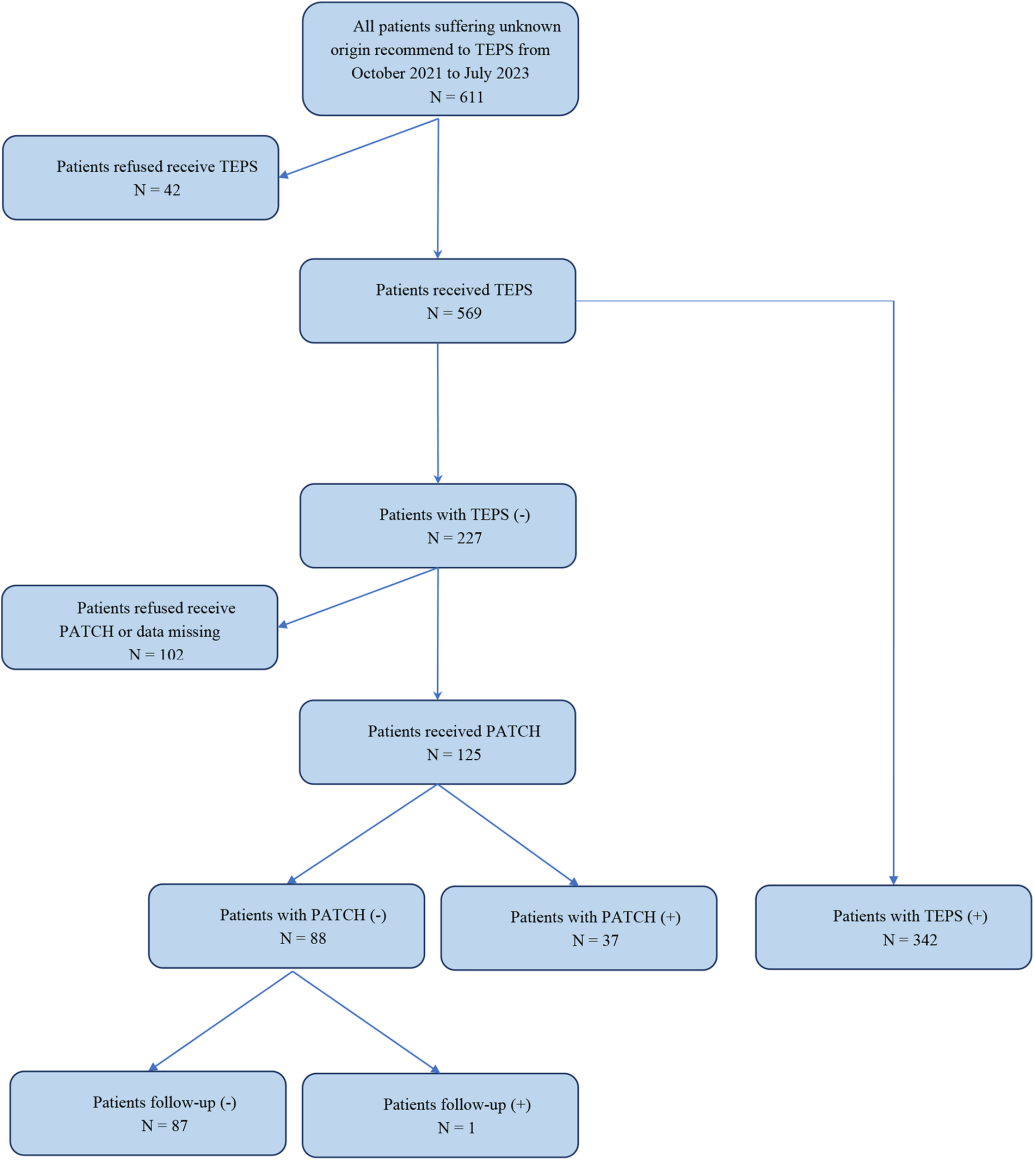
Study flowchart.

A total of 569 patients were initially included in this study; 91 patients who did not exhibit arrhythmia induced by TEPS declined further Patch ECG monitoring, and 11 patients discontinued due to adverse events such as skin allergies were excluded, resulting in the final inclusion of 467 patients in the study (see more details in Figure 1). Among the 467 patients, 211 were males and 256 were females; their ages ranged 10–83 years old, with a mean age of 43.43 ± 17.17 years old. Clinical manifestations contained paroxysmal palpitations in all subjects, dizziness in 50, amaurosis in 2, and syncope in 2 cases. Previous history included smoking in 102 cases, drinking in 207 cases, and radiofrequency ablation in 10 cases. Complications contained coronary heart disease in 44 cases, hypertension in 99 cases, diabetes in 86 cases, cerebrovascular disease in 6 cases, kidney disease in 7 cases, congenital heart disease in 9 cases, hyperlipidemia in 46 cases, and tumors in 3 cases. This study was approved by the Ethics Committee of Henan Provincial People's Hospital.

### 2.2 Research methods

#### 2.2.1 TEPS

DF-5A cardiac electric stimulator and 7F quadrupole esophageal pacing lead were purchased from Suzhou Dongfang Electronic Instrument Factory, Suzhou, China. The study was conducted according to *Noninvasive Cardiac Electrophysiology Diagnostic and Therapeutic Technique-Basic and Clinic* (23).

**Figure 2:**
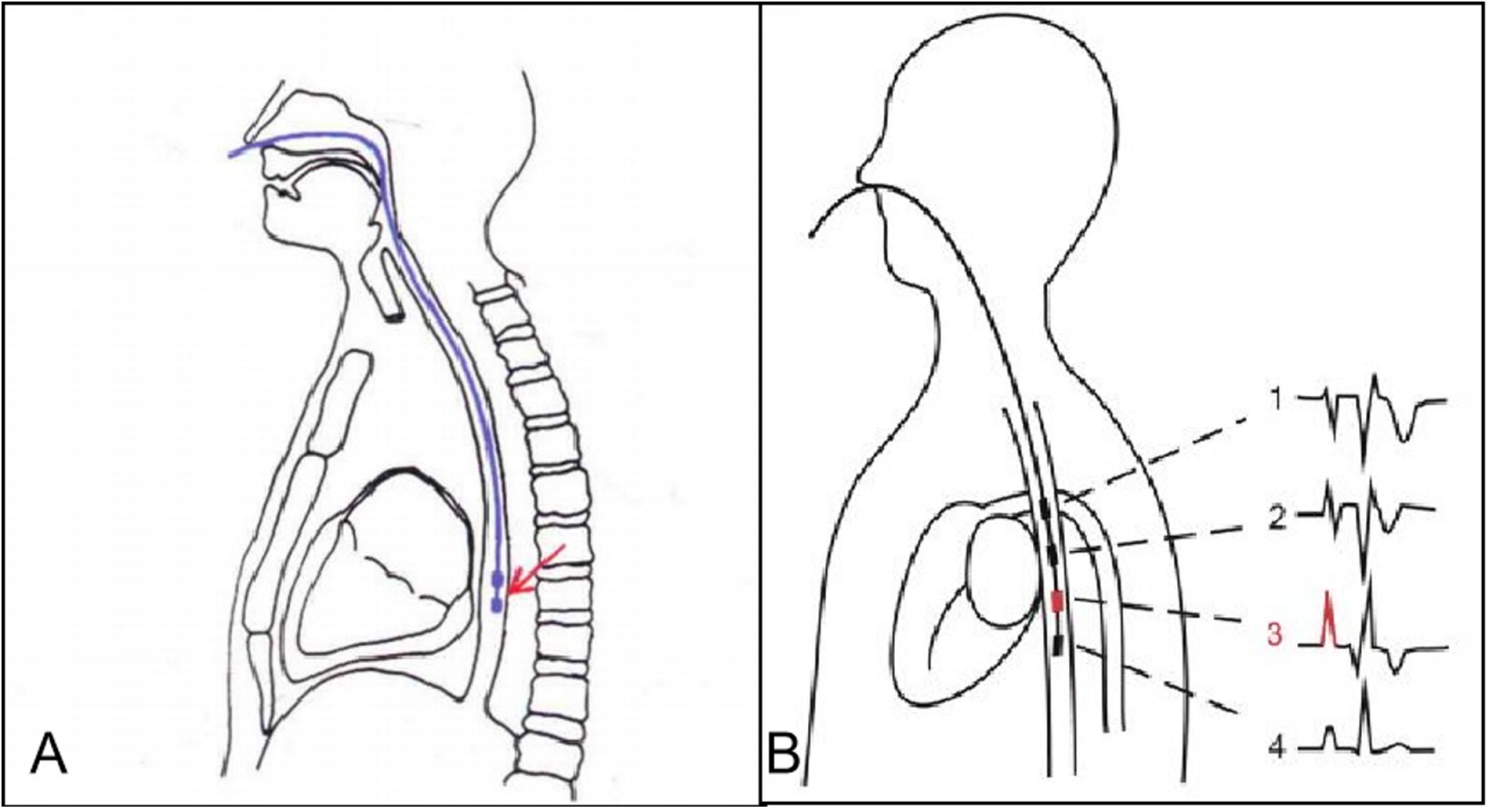
Position of the esophageal pacing lead and the best esophageal electrocardiogram. **(A)** Position of the esophageal pacing lead. **(B)** The best esophageal electrocardiogram is the third one.

**Figure 3:**
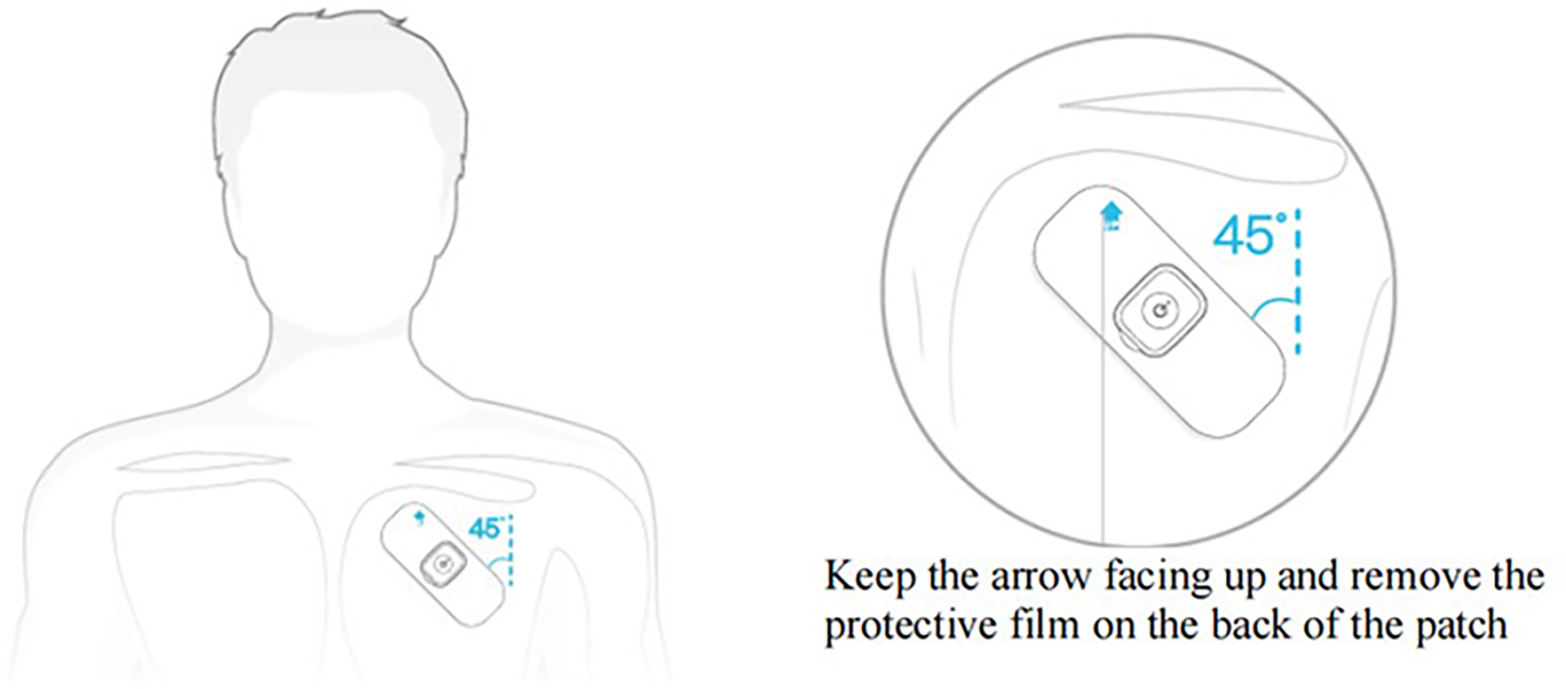
Position of patch.

**Figure 4:**
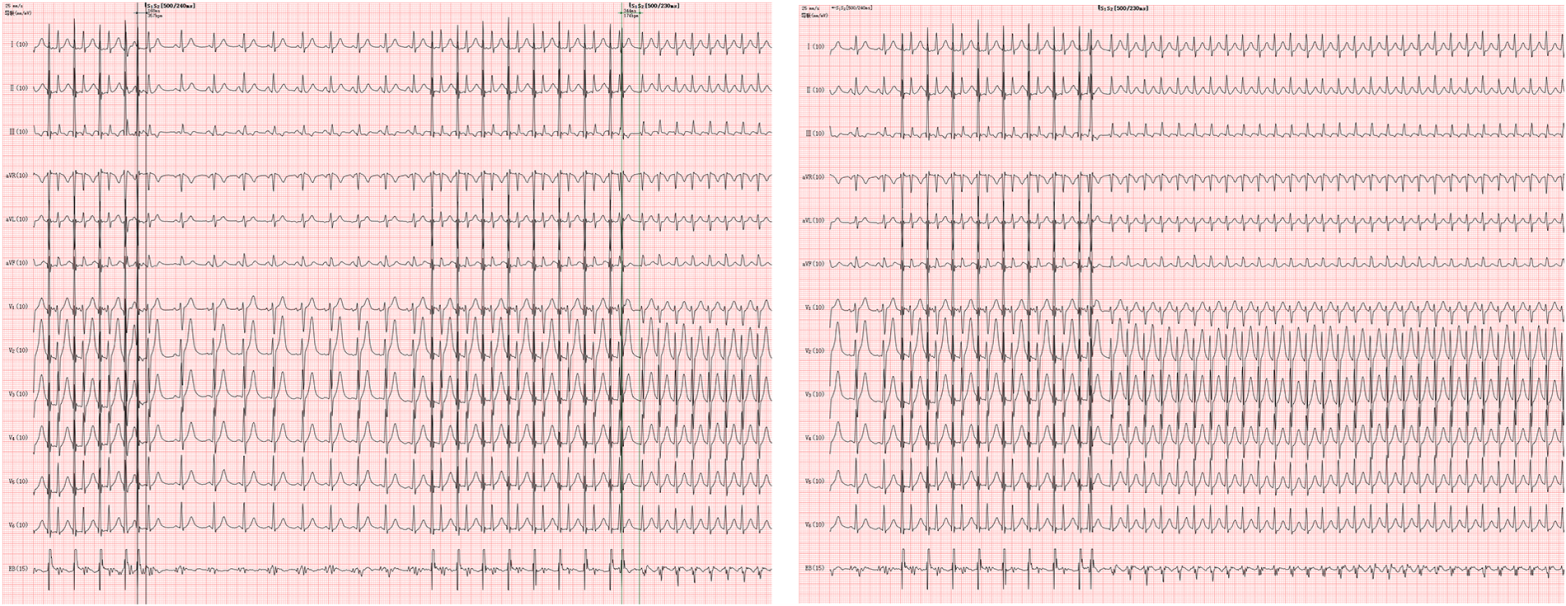
TEPS records of a 32-year-old male patient. The patient was presented with prolonged S2R jump induced by S1S2 stimulation for 176ms in TEPS, and SVT was also induced. Slow-fast atrioventricular nodal reentrant tachycardia (SFAVNRT) was confirmed by esophageal electrocardiogram (EB lead) and body surface electrocardiogram, which was verified by EPS.

The steps were as follows: (1) Contraindications were excluded, and informed consent was obtained; (2) A 7 F esophageal pacing lead was inserted through a nostril into the esophagus with the patient in a supine position without sedation. The depth of insertion of the pacing lead was typically determined based on the distance between the nasal tip and the distal end of the esophageal pacing lead, which was calculated using the formula: (height + 200)/10 + 2 cm (Figure 2A); (3) The positioning of the esophageal pacing leads were further adjusted based on atrial wave (A), ventricular wave (V) size, and A/V ratio observed in the esophageal electrocardiogram. (Figure 2B); (4) After determining atrial pacing thresholds, various pacing maneuvers were performed with minimal stimulus strength required for consistent atrial capture to minimize any discomfort caused by pacing. The pacing maneuvers included single, double, and triple atrial extra stimuli, including RS_2_, S_1_S_2_, S_1_S_2_S_3_ reverse scan, and S_1_S_1_ step-up stimulation, introduced incrementally during sinus rhythm until reaching either atrial or AV node effective refractory period or inducing specific ECG phenomena or arrhythmia. The entire process was recorded in detail; (5) If no arrhythmia was induced and contraindications were ruled out, isoproterenol 0.02–0.04 μg/(kg min) was administered to increase heart rate by 25%–50%, followed by repeating aforementioned stimulation procedures.

Two experienced ECG chief physicians analyzed the TEPS results. If the results were inconsistent, the conclusion was given after discussion. Diagnostic criteria referred to previous results (23, 24).

#### 2.2.2 Patch ECG monitors

Patch ECG monitors were applied to patients who did not exhibit symptom-related arrhythmia induced by TEPS. The device utilized in this study was procured from Hangzhou Proton Technology Co., Ltd., China (model CarePatch ECG-P01).

The steps were as follows: (1) Prior to commencing the measurement, remove any hair from the upper region of the left chest and cleanse the skin with warm water; (2) Attach the Patch ECG monitors to the left chest wall (Figure 3); (3) Provide patients with a symptom diary to document suspected arrhythmia symptoms, including their onset and duration. Depending on when symptoms occurred, patients could then wear it for several weeks.

The results of Patch ECG monitors were independently interpreted by two experienced chief physicians to observe the occurrence of arrhythmia (19, 24). If their conclusions were inconsistent, the final conclusion was given after discussion.

### 2.3 Clinical diagnosis of arrhythmias

Cardiologists determined the occurrence of arrhythmias based on the patient's medical history (attack triggers, frequency, onset and cessation patterns, symptoms during attacks, previous similar episodes and family history, underlying diseases, and medication history), physical examination findings (such as heart rate, rhythm, and abnormal heart sounds), as well as results obtained from TEPS, Patch ECG monitor (23, 24). All patients included in this study underwent TEPS, and induced tachycardia consistent with clinical symptoms could be initially diagnosed and managed clinically with subsequent follow-up. Patients without induced tachycardia used Patch ECG monitors for several weeks, depending on the timing of palpitations onset, and a preliminary diagnosis was possible if palpitations are accompanied by corresponding ECG abnormalities. Clinical follow-up lasted from 11 to 34 months, during which some patients underwent intracardiac electrophysiological study (EPS) for diagnostic clarification and received radiofrequency catheter ablation (RFCA).

### 2.4 Statistical methods

Normality of measurement data was assessed by the Shapiro–Wilk test. Continuous variables were expressed through the mean ± standard deviation or medians with interquartile ranges (IQRs) depending on the distribution. Analysis of the comparisons between the two groups was determined by Student's *t*-test or Mann–Whitney *U* test for normal distributed or non-normal distributed continuous variables. The percentage of cases described the count data. Binary data or sample constituent ratio data was tested by the *χ*^2^ test. The accuracy, sensitivity, specificity, positive predictive value (PPV), negative predictive value (NPV), and 95% confidence interval of all statistical criteria were calculated by the Wald method to evaluate the diagnostic value of all methods. The missing data of the categorical variables were imputed by the dominant category and the continuous variables by the median. *P* < 0.05 was considered significant. Statistical analysis was performed using a commercially available software package (SPSS Statistical Software Version 25.0).

## 3 Results

### 3.1 TEPS results

The TEPS results exhibited that 342 of the 467 patients induced arrhythmias consistent with the same clinical symptoms as the TEPS(+) group, in which the detection rate of supraventricular arrhythmias was high, accounting for 72.38%, and the detection rate of ventricular tachycardia (VT) was low, accounting for only 0.86%. Of the 467 patients, 125 did not induce arrhythmias consistent with clinical symptoms as the TEPS(-) group, with results including completely normal and some specific cardiac phenomena, and some minor arrhythmias (Table 1).

**Table 1:** TEPS results.

TEPS outcomes		Number	Proportion (%)
TEPS(+)	Total (positive)	342	73.23
	SVT	338	72.38
	AVNRT	137	29.34
	AVNRT + AT	9	1.93
	AVNRT + AT + JT	1	0.21
	AVNRT + AVRT	4	0.86
	AVRT	106	22.7
	AT	64	13.7
	AT + AF	3	0.64
	AT + AF + AFL	1	0.21
	Long RP tachycardia	5	1.07
	AF/AFL	8	1.71
	VT	4	0.86
TEPS(-)	Total (negative)	125	26.77
	Dual AV node pathway/multi pathway	20	4.28
	Dual AV node pathway/multi pathway + sinus node dysfunction	1	0.21
	Dual AV node pathway/multi pathway + atrioventricular node dysfunction	1	0.21
	Dual AV node pathway/multi pathway + intermittent pre-excitation pattern	2	0.43
	Dual AV node pathway/multi pathway + atrial echo beat	4	0.86
	Dual AV node pathway/multi pathway + occasional premature beat	11	2.36
	Intermittent pre-excitation pattern	7	1.5
	Intermittent pre-excitation pattern + atrial echo beat	1	0.21
	Atrial echo beat	1	0.21
	Occasional premature beat	58	12.42
	Occasional premature beat + sinus node dysfunction	3	0.64
	Occasional premature beat + sinus node dysfunction + atrioventricular node dysfunction	1	0.21
	Occasional premature beat + atrioventricular node dysfunction	6	1.28
	Occasional premature beat + intermittent pre-excitation pattern	1	0.21
	Occasional premature beat + atrial echo beat	3	0.64
	Normal	2	0.43
	Accelerated atrioventricular node conduction	2	0.43
	Accelerated atrioventricular node conduction/atrial echo beat	1	0.21
Total		467	100.00

SVT, supraventricular tachycardia; AVNRT, atrioventricular nodal reentrant tachycardia; AT, atrial tachycardia; AF/AFL, atrial fibrillation/atrial flutter; VT, ventricular tachycardia; AVRT, atrioventricular reentrant tachycardia; JT, junctional tachycardia.

### 3.2 Patch ECG monitors results

The Patch ECG monitors were worn by 125 TEPS(-) patients for a duration ranging from 2 to 6 weeks; 37 of the 125 patients detected arrhythmias consistent with the same clinical symptoms as the Patch(+) group, in which the detection rate of atrial tachycardia and inappropriate sinus tachycardia was high, accounting for 22.13%. Of the 125 patients, 88 did not detect arrhythmias consistent with clinical symptoms as the Patch(-) group, including completely normal and some specific cardiac phenomena, some minor arrhythmias (Table 2).

**Table 2:** Results of patch ECG monitors.

Patch outcomes	Results	Number	Proportion (%)
PATCH(+)	Total	37	29.60
	AT	9	7.20
	AT + AF	2	1.60
	AT/IST	9	7.20
	PAF	1	0.80
	PAF + PAFL	1	0.80
	PAF + CA	1	0.80
	Long RP tachycardia	1	0.80
	NCT	1	0.80
	IST	2	1.60
	Frequent premature beats	5	4.00
	AVB	2	1.60
	AVB + CA	1	0.80
	CA	2	1.60
PATCH(-)	Total	88	70.40
	Normal	26	20.80
	Occasional premature beat	57	45.60
	Occasional premature beat + SB	1	0.80
	Intermittent pre-excitation pattern	2	1.60
	SB	2	1.60
Total		125	100.00

IST, inappropriate sinus tachycardia; AT, atrial tachycardia; PAF, paroxysmal atrial fibrillation; PAFL, paroxysmal atrial flutter; CA, cardiac arrest; NCT, narrow complex tachycardia; AVB, atrioventricular block; SB, sinus bradycardia.

### 3.3 Clinical diagnostic results of TEPS in the diagnosis of all patients suffering unexplained palpitations

A total of 379 patients were diagnosed with arrhythmia according to clinical evaluation and follow-up, and a total of 167 patients underwent EPS and RFCA, including 157 cases of SVT, 8 cases of paroxysm atrial fibrillation and atrial flutter (PAF/PAFL), and 2 cases of VT. Among 467 participants with TEPS, results were positive in 342, for an accuracy of 91.65% (95% CI, 89.14%–94.16%). Meanwhile, TEPS showed good sensitivity (89.97%, 95% CI, 86.95%–93.00%), specificity (98.86%, 95% CI, 96.65%–100.00%), and PPV (99.71%, 95% CI, 99.14%–100.00%). However, the NPV of TEPS did not show good performance (69.60%, 95% CI, 61.54%–77.66%). Details of the diagnostic testing are provided in Table 3.

**Table 3:** Accuracy, sensitivity, specificity, PPV, and NPV of TEPS.

Item	Event	Percentage	95% CI
Accuracy	428/467	91.65	89.14–94.16
Sensitivity	341/379	89.97	86.95–93.00
Specificity	87/88	98.86	96.65–100.00
Positive predictive value	341/342	99.71	99.14–100.00
Negative predictive value	87/125	69.60	61.54–77.66

TP, true positive; FP, false positive; FN, false negative; TN, true negative.

The table describes the diagnostic effect of TEPS for all patients. Confidence interval was calculated by Wald method.

Accuracy = (TP + TN)/(TP + FP + FN + TN); Sensitivity = TP/(TP + FN); Specificity = TN/(TN + FP); Positive predictive value = TP/(TP + FP); Negative predictive value = TN/(TN + FN).

### 3.4 Clinical diagnostic results of Patch ECG monitors in the diagnosis of patients with negative results of TEPS

Among 125 participants who accepted Patch ECG monitors, the result was positive in 37, with an accuracy of 99.20% (95% CI, 97.64%–100.00%). The sensitivity of Patch ECG monitors for the patients was 97.37% (95% CI, 92.28%–100.00%). Both specificity and PPV were 100.00% (95% CI, 100.00%–100.00%). NPV was 98.86% (95% CI, 96.65%–100.00%). Details of the diagnostic testing are provided in Table 4.

**Table 4:** Accuracy, sensitivity, specificity, PPV, and NPV of patch ECG monitors in patients with negative result of TEPS.

Item	Event	Percentage	95% CI
Accuracy	124/125	99.20	97.64–100.00
Sensitivity	37/38	97.37	92.28–100.00
Specificity	87/87	100.00	100.00–100.00
Positive predictive value	37/37	100.00	100.00–100.00
Negative predictive value	87/88	98.86	96.65–100.00

TP, true positive; FP, false positive; FN, false negative; TN, true negative.

The table describes the diagnostic effect of Patch ECG monitors in patients with negative result of TEPS. Confidence interval was calculated by Wald method.

Accuracy = (TP + TN)/(TP + FP + FN + TN); Sensitivity = TP/(TP + FN); Specificity = TN/(TN + FP); Positive predictive value = TP/(TP + FP); Negative predictive value = TN/(TN + FN).

### 3.5 Clinical diagnostic results of TEPS combined with Patch ECG monitors in the diagnosis of all patients suffering unexplained palpitations

The combined diagnosis method showed better performance than TEPS alone. The accuracy (99.57%, 95% CI, 98.98%–100.00%), sensitivity (99.74%, 95% CI, 99.22%–100.00%), and NPV (98.86%, 95% CI, 96.65%–100.00%) were significantly higher than TEPS. Meanwhile, specificity (98.86%, 96.65%–100.00%) and PPV (99.74%, 99.22%–100.00%) still have a good performance. Details of the diagnostic testing are provided in Table 5.

**Table 5:** Accuracy, sensitivity, specificity, PPV, and NPV of combined diagnosis method.

Item	Event	Percentage	95% CI
Accuracy	465/467	99.57	98.98–100.00
Sensitivity	378/379	99.74	99.22–100.00
Specificity	87/88	98.86	96.65–100.00
Positive predictive value	378/379	99.74	99.22–100.00
Negative predictive value	87/88	98.86	96.65–100.00

TP, true positive; FP, false positive; FN, false negative; TN, true negative.

The table describes the diagnostic effect of combined diagnosis method for all patients. Confidence interval was calculated by Wald method.

Accuracy = (TP + TN)/(TP + FP + FN + TN); Sensitivity = TP/(TP + FN); Specificity = TN/(TN + FP); Positive predictive value = TP/(TP + FP); Negative predictive value = TN/(TN + FN).

## 4 Discussion

Current guidelines recommend ambulatory ECG monitoring as the most vital tool for diagnosing palpitations of unknown origin (4, 25). As a type of ambulatory ECG monitoring, Patch ECG monitors recently have been widely used in clinical practice because of their advantages of being non-invasive, having a prolonged observation period, and being relatively reliable recording. This device effectively records a wide array of abnormal ECG signals in the most natural state of patients, significantly enhancing the accuracy of disease diagnosis (26–33).

Previous studies indicate a duration of up to 1–2 weeks of Patch ECG monitoring yields a high rate of arrhythmia identification, with the diagnostic rate of palpitations during 1–4 weeks of Patch ECG monitoring varied between 70% and 85% (4, 33). Diagnostic efficacy is limited primarily by the need to wait for symptom recurrence (4). The use of single or three-channel formats in Patch ECG monitors also makes it challenging to discern specific types of captured arrhythmias. For symptomatic patients with challenging diagnosis, and a strong suspicion of arrhythmia, current recommendation suggest considering Loop record implantation or intracardiac EPS (2). However, ESP may not be suitable for all individuals due to its invasiveness, peripheral vascular issues, cardiac complications, the longer recovery period, and other factors.

In comparison to EPS, TEPS was selected for patient evaluation in our study due to its relatively non-invasive, economical, convenient, and acceptable accuracy. Current guidelines recommend esophageal electrocardiogram as a useful reference point for distinguishing narrow QRS tachycardia, demonstrating its clinical application value (19). Reported inducibility rates with this method for supraventricular tachycardia range from 73% to 98.5% (34–36), and the results regarding inducibility and tachycardia mechanism showing excellent correlation with findings on subsequent EPS (12, 37). Among the 342 patients in the TEPS(+) group, a total of 165 patients underwent EPS and RFCA, including 157 cases of SVT, 6 cases of PAF/PAFL, as well as 2 cases of VT. Apart from providing a specific diagnosis for SVT, TEPS demonstrated high diagnostic accuracy across different types of inducibility. According to our study, TEPS exhibits good overall diagnostic efficacy and holds certain diagnostic values for suspected paroxysmal supraventricular tachycardia in patients (Figure 4). This conclusion aligns with previous studies. However, it is important to note that the negative predictive value is low, and approximately 30.4% of patients may be missed, leading to potential diagnostic and therapeutic bias.

Since the combination of Patch ECG monitors and TEPS in the diagnosis of palpitations of unknown origin may yield accurate diagnostic results, their combination was employed in this study. Depending on the time of onset of palpitations, patients without induced tachycardia in TEPS required several weeks of Patch ECG monitoring. There are several findings from the combined diagnosis.

**Figure 5:**
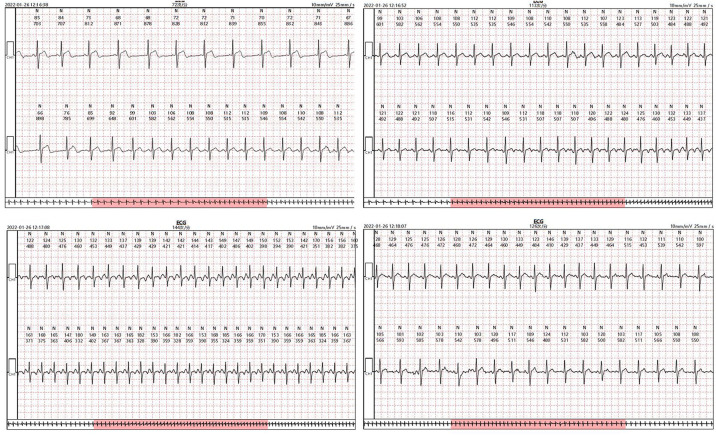
Patch ECG fragments of a 17-year-old male patient diagnosed with inappropriate sinus tachycardia. The Patch ECG monitors recorded the patient's spontaneous symptoms during a resting state, aiding in the detection of misdiagnoses by TEPS.

First, the results demonstrate that Patch ECG monitors can effectively detect certain types of palpitations such as autonomic atrial tachycardia, inappropriate sinus tachycardia (or postural tachycardia) (Figure 5), paroxysmal atrial fibrillation, atrial flutter, and some bradyarrhythmia associated with automaticity, which were missed by TEPS. According to the follow-up, two cases underwent PAF/PAFL radiofrequency ablation and two cases underwent pacemaker implantation in the Patch(+) group. Due to the global coronavirus epidemic during the study period, autonomic dysfunction or underlying neo-coronavirus infection, stress, and anxiety could explain the more challenging-to-diagnose arrhythmias identified in this study, which aligns with findings from previous studies (38–40). Thus, Patch ECG monitors can effectively identify patients who have been underdiagnosed and prevent treatment delays.

**Figure 6:**
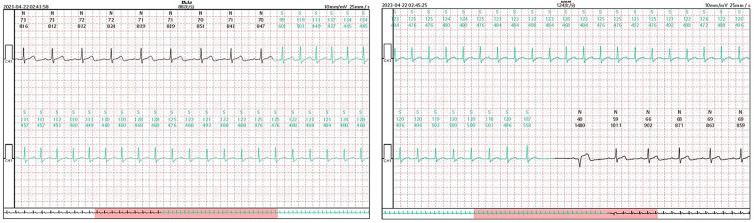
Patch ECG fragments of a 45-year-old female patient diagnosed with Supraventricular tachycardia. TEPS detected only dual atrioventricular nodal pathways, while the occurrence of tachycardia was documented by the Patch ECG monitors in the fourth week.

Second, only one patient with atrioventricular nodal reentrant tachycardia was missed; in this case, TEPS detected only dual atrioventricular nodal pathways, and the occurrence of tachycardia was documented by the Patch ECG monitors in the fourth week (Figure 6). This finding suggests the diagnosis of atrioventricular reentrant tachycardia and atrioventricular reentrant tachycardia could be ruled out if the TEPS results are non-inducible and there is no jump phenomenon, pre-excitation, or atrial echo beat. This is consistent with the study of Michel et al. (9).

Third, although typical symptoms were detected in some patients, no corresponding arrhythmia was detected in the result analysis, and the diagnosis of arrhythmia-related diseases was also excluded; it was recommended to seek medical treatment in the departments of respiration and neurology.

Our results exhibited that the consistency, accuracy, sensitivity, and negative predictive value of the combined diagnosis of the two methods were higher than that of the TEPS alone, suggesting that the Patch ECG monitors combined with TEPS can elevate the diagnostic efficiency of palpitations of unknown origin. The reasons may be as follows: The combined application of Patch ECG monitors and TEPS can make up for each other's shortcomings. For example, TEPS has a good diagnostic value in inducing tachycardia involving a reentry mechanism and triggering mechanism using atrial program stimulation and drug stimulation. It provides very detailed information for the definite diagnosis of arrhythmia. In addition, it is undeniable that a substantial number of patients display negative TEPS findings. Before the implementation of Patch ECG monitors, the identification of these patients resided in a “murky territory,” thereby intensifying the strain on patients’ diagnostic clarity, psychological state, and financial stability. Our study utilized Patch ECG monitors to analyze patients in the TEPS(-) group, bridging the diagnosis gap. The detection rate of inappropriate sinus tachycardia, automatic atrial tachycardia, paroxysmal atrial fibrillation, and atrial flutter can be improved by Patch ECG monitors, and thus the diagnostic efficiency can be improved. The combined diagnosis method is conducive to diminishing the occurrence of excessive medical treatment and alleviating the patient's medical pressure and psychological pressure.

The researcher's hospital has a large volume of patient visits and high levels of patient compliance. Moreover, the hospital possesses ample relevant diagnostic expertise to investigate supplementary research on initial TEPS-negative diagnoses. Based on the above research advantages, we explored the possibility of combining TEPS with Patch ECG monitors, which reduces the possibility of missed diagnosis of the disease to a certain extent on the basis of lowering the patients’ medical cost and pain of examination and provides a feasible solution for the future diagnosis of clinical palpitation.

In the future, we could also explore the use of wearable ECG devices such as Apple Watch or ALIVECOR/Kardiamobile to replace Patch ECG monitors, which could extend the observation period and improve patient compliance, thereby improving the disease diagnosis rate (41, 42). Moreover, we will investigate the scoring system and further explore the implementation of the corresponding diagnostic methods to improve the degree of diagnostic standardization.

## 5 Limitations

The study was a retrospective cohort study, limiting the ability to address issues related to patient selection and sample size calculation through experimental design. Further studies should use randomized controlled trials. In addition, the diagnosis of the TEPS(+) group was established based on a comparison between induced tachycardia and patient-reported symptoms. No further Patch ECG monitors were employed within the TEPS(+) group, and only a subset of patients consented to undergo a more rigorous verification process involving EPS. Meanwhile, some patients of TEPS(-) group declined to undergo Patch ECG monitors due to milder symptoms and adverse reactions associated with wearing, which had an impact on the study findings. This influenced the overall outcomes of the study, potentially skewing the data and making it less representative of the broader population with similar symptoms. These factors should be considered when interpreting the results and designing future research in this field.

## 6 Conclusion

Our findings support the efficient diagnostic capabilities of TEPS in identifying paroxysmal supraventricular tachycardia using various programmed stimuli. In addition, combining Patch ECG monitors and TEPS can effectively reduce missed diagnoses associated with TEPS and enhance the diagnostic efficiency of palpitations of unknown origin.

## Data Availability

Due to patient privacy concerns, the raw data supporting the conclusions of this article will be made available after approved by the Ethics Committee of Henan Provincial People's Hospital.
