# Supplementary material for: Wearable Patch ECG monitors and transesophageal electrophysiological study for diagnosing palpitations of unknown origin
Source: Front Cardiovasc Med. 2024 Nov 13;11:1469108. doi: 10.3389/fcvm.2024.1469108 (PMC11598447; doi:10.3389/fcvm.2024.1469108)
Supplement: Supplementary file 1 [file Table1.doc]

Supplement table 1 Characteristics in patients accept teps.

Items	Types	All cohort	Teps (-)	Teps (+)	P value for normality	P value for comparison	
Age		46.00(30.00-56.00)	45.00(27.00-56.00)	47.00(31.00-56.00)	<0.001	0.365	
Gender 	Male	211(45.18)	55(44.00)	156(45.61)	-	0.756	
	Female	256(54.82)	70(56.00)	186(54.39)			
	Total	467(100.00)	125(100.00)	342(100.00)			
